# Emerging Mechanisms of Skeletal Muscle Homeostasis and Cachexia: The SUMO Perspective

**DOI:** 10.3390/cells12040644

**Published:** 2023-02-17

**Authors:** Bushra Khan, Luis Vincens Gand, Mamta Amrute-Nayak, Arnab Nayak

**Affiliations:** Institute of Molecular and Cell Physiology, Hannover Medical School, Carl-Neuberg-Str. 1, 30625 Hannover, Germany

**Keywords:** sarcomere, muscle atrophy, cachexia, muscle metabolism, chromatin signaling, ubiquitination, ubiquitin-like modifiers, SUMO-specific isopeptidase

## Abstract

Mobility is an intrinsic feature of the animal kingdom that stimulates evolutionary processes and determines the biological success of animals. Skeletal muscle is the primary driver of voluntary movements. Besides, skeletal muscles have an immense impact on regulating glucose, amino acid, and lipid homeostasis. Muscle atrophy/wasting conditions are accompanied by a drastic effect on muscle function and disrupt steady-state muscle physiology. Cachexia is a complex multifactorial muscle wasting syndrome characterized by extreme loss of skeletal muscle mass, resulting in a dramatic decrease in life quality and reported mortality in more than 30% of patients with advanced cancers. The lack of directed treatments to prevent or relieve muscle loss indicates our inadequate knowledge of molecular mechanisms involved in muscle cell organization and the molecular etiology of cancer-induced cachexia (CIC). This review highlights the latest knowledge of regulatory mechanisms involved in maintaining muscle function and their deregulation in wasting syndromes, particularly in cachexia. Recently, protein posttranslational modification by the small ubiquitin-like modifier (SUMO) has emerged as a key regulatory mechanism of protein function with implications for different aspects of cell physiology and diseases. We also review an atypical association of SUMO-mediated pathways in this context and deliberate on potential treatment strategies to alleviate muscle atrophy.

## 1. Introduction

Skeletal muscle is an astounding organ constituting over 650 muscles accounting for approximately 40% of total body mass and up to 30% of basal energy expenditure [1]. The skeletal muscle is made up of muscle cells, comprising several myofibrils. The myofibrils contain linear arrays of contractile units, the sarcomere. The precisely organized sarcomeres consist of myosin-based thick filaments and actin-based thin filaments, which are further supported by macromolecular proteins, such as titin and nebulin along the filaments and protein complexes residing in Z-bands. The region between two Z-bands define the length of one sarcomeric unit, which is approximately 2.2 μm in the case of human skeletal muscle. According to the sliding filament model [2], the thick and thin filaments slide past each other, whereby the ATP-driven cyclical interaction between the actin and myosin filaments causes the muscle shortening, generating the force necessary for movements at the molecular as well as organismic level. Muscle contraction upon neural stimulation is responsible for driving the movements, maintaining balance, regulating body posture, breathing, and controlling the body temperature. Skeletal muscles are also critical reservoirs of nutrient including glycogen and amino acids. While muscle glycogen serves as energy stores for muscles itself, the amino acids are released as energy sources for other tissues as per requirement [3]. In insulin-stimulated condition, skeletal muscles can consume nearly 75% of circulating glucose [4]. Thus, muscle functions are intertwined with the basic metabolism and overall health of animals. Various factors, including mechanical, oxidative, nutritional stresses, and cytokines, critically regulate distinct signaling pathways in skeletal muscles and modulate a balance between muscle structure breakdowns and muscle growth. Deregulation of this finely tuned protein and organelle turnover mechanisms can result in muscle disorders including muscle wasting conditions with an impact on muscle contractile ability and thereby force generation.

Sarcopenia and cachexia represent common types of muscle wasting disorders. Sarcopenia is an age-related progressive loss of skeletal muscle mass, strength, and physical performance in 14% of people aged 60 years and above and up to 53% of people aged 80 years and above [5]. The most common drivers of sarcopenia include neurodegeneration, hormonal changes, activation of inflammatory pathways, increased insulin resistance and a decline in physical activity. Physical exercise and high-protein diets have been shown beneficial in managing and even preventing sarcopenia [6,7]. On the other hand, cachexia is a complex debilitating muscle-wasting syndrome associated with several diseases, including cancer, congestive heart failure, amyotrophic lateral sclerosis (ALS), chronic obstructive pulmonary disease (COPD), diabetes, acquired immunodeficiency syndrome (AIDS), etc. [8,9]. Strikingly, cachexia affects about 80% of cancer patients, causing severe loss of muscle mass by up to 30–40% of their pre-illness level [10,11]. By secreting various pro-inflammatory cytokines, cancer shifts the balance of muscle metabolism toward the catabolic direction and thus induces cachexia, also termed as cancer-associated or cancer-induced cachexia (CIC) [12]. Although it is arguable whether CIC is an inadvertent side effect of cancers, few reports suggested that cancer promotes the breakdown of muscle proteins to meet the high energy demands of cancer cells, thereby accelerating the tumor progression process [13,14]. In addition to CIC, several chemotherapeutic treatment regimens also induce cachexia, thereby exacerbating already existing debilitating conditions [15]. 

Cachexia (“*Kakos*” means bad and “*hexis*” means condition in Greek) is one of the oldest known human conditions reported as early as 4th century BC by Hippocrates, who described it as, *“the flesh is consumed and becomes water. The shoulders, clavicles, chest, and thighs melt away. The illness is fatal.”* [16]. The term “cancerous cachexia” was coined by John Zachariah in 1858 [17] to describe the muscle-wasting syndrome linked with malignancy. Since then, this syndrome was overlooked in the clinics as the focus was about curing the primary illness i.e., cancer, to which cachexia is associated. As a result, there was no working definition of cachexia until very recently. In 2011, cachexia was formally defined as, “a multifactorial syndrome characterized by ongoing loss of skeletal muscle (with or without loss of fat mass) that cannot be fully reversed by conventional nutritional support and leads progressive functional impairment” [18]. Reduced life quality and high mortality rate linked to the cachectic condition has brought the attention of the researchers to this secondary yet severe coexisting condition. Contrary to sarcopenia, the progression of CIC is rather rapid and a loss in body mass of more than 5% over six months is currently designated as the commencement of cachectic state [18]. Loss of skeletal muscle associated with CIC, lowers the protein and energy availability throughout the body, causing delayed recovery from illness, alteration in resting metabolic rate, anabolic resistance, physical disability, poorer quality of life, and even death when respiratory muscles are too weak to support the breathing. 

Precise assembly and accurate functioning of the sarcomeric proteins is prerequisite for the proper contractile function of muscle. Sarcomere/myofibrillar disarray is a typical feature of myopathies, mainly caused by dysfunction of the contractile proteins. Apart from cancer, the chemotherapeutic drugs used to cure cancer were shown to affect sarcomere organization and exacerbate the muscle atrophy [19]. Both CIC and chemotherapy-induced cachexia are associated with distinct metabolic derangements [12]. However, the knowledge about a master regulator of cachexia remains elusive. The lack of sufficient knowledge is also reflected in the high mortality rate of a staggering 30% of cancer patients that is associated with the cachexia rather than due to the cancer itself [20,21]. There is an urgent unmet need for the in-depth understanding and development of therapeutic interventions to curb this debilitating condition.

CIC is a multi-organ metabolic disorder, primarily disrupting muscle function by destabilizing a steady-state balance involving muscle mass growth (anabolic) versus muscle breakdown (catabolic). Protein posttranslational modification by the SUMO (Small Ubiquitin-like Modifier) pathway regulates various aspect of cell function including proteostasis, and thus has important physiological and pathophysiological implications [22]. Evidence regarding the functional link between SUMOs with muscle cell function and muscle atrophy is rather scarce and is one of the evolving areas of biology. This review primarily focusses on the current knowledge of how the skeletal muscle homeostasis is disturbed in cancer-induced cachexia. We will discuss new emerging players, such as the SUMOylation pathway in cachexia, and implications of novel insights for potential pharmacological interventions.

## 2. Cachexia Affects Muscle Cell Signaling Mechanisms and Metabolism

### 2.1. Cachexia and Muscle-Specific E3 Ubiquitin Ligases

Muscle atrophy is one of the prominent hallmarks of CIC, pertaining to the deregulation of muscle protein anabolic and catabolic signaling pathways. The muscle is often targeted during the tumor progression. Tumors secrete a multitude of pro-inflammatory cytokines into their microenvironment and into the circulatory system, e.g., tumor-necrosis factor α (TNF-α), interleukin (IL)-1 and -6, interferon-γ (IFN-γ), proteolysis-inducing factor (PIF), and tumor growth factor β (TGF-β) among others [21,23]. The metastatic tumor-secreted molecules particularly TNF-α and IL-1 induced catabolism in muscle cells in a p38 MAPK (mitogen-activated protein kinase)- and nuclear factor ‘kappa-light-chain-enhancer’ of activated B-cells (NF-κB)-dependent manner [24]. Activation and nuclear translocation of NF-κB resulted in increased expression of the muscle-specific E3 ubiquitin ligase muscle RING finger protein 1 (MuRF1) [25,26]. MuRF1-mediated ubiquitination of many myofibrillar proteins, particularly myosin heavy chain (MHC), myosin-binding protein C (MyBP-C), and myosin light chains 1 and 2 (MLC1 and MLC2), led to their proteasome-dependent degradation [26,27]. Atrogin1/MAFbx (muscle atrophy F-box) is another type of major muscle-specific E3 ubiquitin ligase upregulated in CIC [28]. Both atrogin1 and MuRF1 were shown to be upregulated in several CIC models [29,30,31]. Additionally, PIF exposure to murine C2C12 myotubes resulted in IκBα degradation and nuclear accumulation of NF-κB. This was followed by increased proteasomal activity and protein expression of the 19S subunits mss1 and p42 and the ubiquitin conjugating enzyme E2 (14K) [32]. Along the same line of observation, the expression of C2 and C5 subunits of the proteasome [33], as well as the ATPase subunit mss1 [34], were increased in CIC. Moreover, atrogin1 induced degradation of MyoD, thus preventing myogenic differentiation and influencing the muscle regeneration [35]. Targeting MuRF1 by small molecule inhibitors in skeletal myotubes and mice models has shown as a promising strategy towards improving skeletal muscle function as well as attenuating muscle atrophy [36,37,38]. Very recently, another muscle-specific E3 ubiquitin ligase, UBR2 (Ubiquitin Protein Ligase E3 Component N-Recognin 2) was reported as a novel player involved in CIC [39]. UBR2 was upregulated through the p38β MAPK–C/EBPβ (CCAAT/ enhancer binding protein beta) signaling pathway and caused selective degradation of MHC, particularly in fast-twitch muscle as a mechanism towards establishing cachexia in diverse cancer types. In a cachexia mice model as well as in cell culture models, UBR2 was shown to interact and ubiquitinate specifically MHC-IIb and -IIx isoforms and target them for proteosomal degradation, resulting in a loss of muscle mass. Interestingly, myosin isoforms, such as MHC-I and MHC-IIa, and other sarcomeric contractile proteins, troponin, tropomyosin, and tropomodulin remained unchanged by UBR2 upregulation. Skeletal muscle-specific knockout of UBR2 improved fast-twitch muscle mass and function in tumor-bearing mice. A similar UBR2-regulated mechanism was found to be conserved in humans, as part of the observation from mice was also validated in *rectus abdominis* skeletal muscle isolated from early stage of human cancer patients.

### 2.2. Cachexia and Akt-mTOR Signaling

The loss of skeletal muscle mass in cachexia occurs partly due to a decrease in protein synthesis, which stems either from a reduced supply of amino acids and energy or a lack of anabolic factors that stimulate the cellular processes of muscle protein production [40]. Multiple upstream regulators that influence muscle protein synthesis prompt the mammalian target of the rapamycin (mTOR) pathway. Insulin and insulin-like growth factor (IGF1) are the two major anabolic factors that feed into the mTOR pathway by activating phosphoinositide 3-kinase (PI3K) which in turn recruits and phosphorylates protein kinase B (Akt), to promote muscle protein synthesis. Akt phosphorylation is required for activation of the mammalian target of rapamycin complex1 (mTORC1) through phosphorylation of tuberous sclerosis 2 (TSC2). Phosphorylation of TSC2- a negative regulatory component in mTOR pathway- inactivates TSC2 and thus stimulates mTOR pathway and protein anabolism [41]. On the contrary, activated Akt can inhibit protein degradation by repressing the forkhead box protein O (FoxO) family of transcription factors, which are originally responsible for the activation of atrogin1 and MuRF1 ubiquitin ligases. Moreover, Akt-mediated phosphorylation of GSK3β leads to the release of GSK3β-repressed eukaryotic translation initiation factor 2B (eIF2B), thereby promoting protein synthesis. Inactivation of GSK3β also relieves the un-phosphorylated form of sarcomeric protein nebulin to interact with N-WASP (Neural Wiskott-Aldrich Syndrome Protein) and initiate actin polymerization for myofibril assembly. In CIC, upregulation of dsRNA-dependent protein kinase (PKR) leads to phosphorylation of eIF2, which prevents its conversion to the active GTP-bound form, thereby repressing muscle protein synthesis [42,43]. A diminished Akt-mTORC1 signaling with increased protein catabolism was associated with CIC [44]. Whereas skeletal muscle-specific activation of the Akt- mTORC1 pathway in C26-bearing cachectic mice could revert muscle wasting by suppressing protein degradation and normalizing the muscle transcriptome [44]. 

Pro-atrophic factors TNF-induced impaired phosphorylation and deactivation of Akt also resulted in FoxO3 activation thus enhancing the expression of *atrogin1* and *MuRF1* genes [45], as well as inducing autophagosome formation through stimulating Bnip3 (BCL2 interacting protein 3) upregulation [46,47]. Along similar lines, overexpression of a TGF superfamily member protein, myostatin, was sufficient to induce atrogin1 expression and lowered Akt signaling [48] by decreasing Akt phosphorylation. In CIC, the expression and activity of myostatin is increased in response to pro-inflammatory cytokines like TNFα. This enables the activation of Smad2/3 transcription factors, thereby allowing the expression of pro-atrophic genes and an upregulation of the ubiquitin-mediated proteosomal degradation pathway. 

Further studies have highlighted the role of amino acids in regulating protein synthesis via the mTOR pathway in a muscle fiber-type dependent manner. Branched-chain amino acids and arginine suppress protein hydrolysis in fast-twitch muscle fibers by inhibiting the expression of *atrogin1* and *MuRF1* via the mTOR pathway in a nitric oxide (NO)-dependent manner. Additionally, these amino acids stimulate mTOR phosphorylation, which in turn stimulates protein synthesis by downstream effector proteins, namely eukaryotic translation initiation factor 4E (eIF4E)-binding protein 1 (4E-BP1) and p70S6 kinase [49,50,51]. Another pathway that influences mTOR signaling is the AMP-activated protein kinase (AMPK) pathway. In CIC, AMPK activity is increased as a result of increased AMP/ATP ratio [52,53], leading to the phosphorylation of raptor, a component of mTORC1 complex, with several implications, ranging from impaired TCA cycle activity to reduced protein synthesis [54].

### 2.3. Cachexia, Glucose Uptake & Metabolism

Following blood glucose level increase after a meal, pancreatic beta cells secrete insulin, necessary for the transport of glucose into insulin-sensitive tissues such as muscle and mTOR signaling-dependent suppression of the proteolysis [55]. Increased insulin resistance, reduced insulin secretion from the pancreas, and glucose intolerance were observed in patients with pancreatic cancer [56] as well as in animal models, and are associated with higher cancer mortality in a longitudinal study [57]. Insulin resistance and weight loss in the CIC mouse model were partially improved by treatment with an insulin-sensitizing agent, rosiglitazone [58], suggesting that targeting insulin resistance could be a useful way to deal with CIC. Insulin resistance can also arise due to the inhibition of Akt substrate 160 phosphorylation, leading to impaired GLUT4 translocation and glucose uptake, a characteristic feature of the cachexia [59]. The connection of insulin resistance with CIC appears rather complex, as increased glucose uptake was also observed in the cachectic myotubes [60]. Inducing cachexia with conditioned medium from murine CT26 carcinoma cells additionally decreased oxygen consumption, altered mitochondria metabolism, and increased lactate production. This appears to be the result of an enhanced glycolysis [60].

### 2.4. Cachexia and the Vicious Cori Cycle

The Cori cycle or the lactic acid cycle is a basic biochemical mechanism by which lactate produced in muscles is transported to the liver, where lactate is converted back into glucose and returned to the muscles [61]. Under normal physiological conditions, lactate produced through anaerobic glycolysis in skeletal muscle is transferred to the liver and converted to glucose by gluconeogenesis. This glucose is released into circulation, taken up by the muscle, and further metabolized back into lactate. In cancer, elevated metabolic needs of cancer cells lead to an increase in glucose uptake and alteration of the glucose utilization program, i.e., overdriving the anaerobic glycolysis for rapid generation of ATP. This results in higher lactate production in cancer cells, a process known as the “Warburg effect” [62]. Overdriving anaerobic glycolysis in turn accelerates Cori cycle rate and thereby realizes the increased glucose demand of the cancer cell. Cachectic myotubes also exhibited similar metabolic patterns to cancer cells i.e., increased lactate production and increased rate of Cori cycle [60]. Besides glucose, triacylglycerol breakdown into free fatty acids in the liver serves as a source of ketone bodies, another source of energy. Interestingly, while most cancer cells have impaired ketone body metabolism, cachectic muscle however retains the ability to metabolize ketone bodies [63] and produces energy from this additional source. Exploiting this mode of the metabolic pathway, particularly interfering with higher Cori cycle rate, is increasingly being viewed as a possible strategy to interfere with cachexia, as discussed in a later section (intervention and treatment strategies). One example of this is the glucose analog 2-Deoxy-D-glucose (2-DG) which can suppress glycolysis by competing with glucose to bind hexokinase (HK), the first rate-limiting enzyme of glycolysis [64]. Through this mechanism, 2-DG can reprogram the glucose metabolism in various metabolism-associated disorders, including breast cancer [65]. In C26 tumor-bearing cachectic mouse model, 2-DG treatment promoted ketone body utilization in cachectic muscle and reduced liver gluconeogenesis and the Cori cycle. By blocking glycolysis, 2-DG forces the cell to use an alternative fuel i.e., ketone bodies, for their energy requirement. This was accompanied by a significant increase in acetyl-coenzyme A (Ac-CoA, the common integrator of protein, lipid, and carbohydrate metabolism), *ACAT1* expression (involved in ketone body utilization), and ATP levels in the skeletal muscle. Thus, while cancer cell could not successfully adapt to this metabolic challenge, cachectic myotubes could use ketone bodies as alternative energy source with lower lactate level. Besides carbohydrate metabolism, 2-DG treatment also suppressed MuRF1 and atrogin1 expression. With all these metabolic and transcriptional alterations, 2-DG, by influencing the Cori cycle, could attenuate muscle atrophy in a mouse model of cachexia. 

### 2.5. JAK/STAT Pathway and Cachexia

Janus kinase (JAKs) and signal transducer and activator of transcription (STATs) factors belonging to the tyrosine kinase family members are used by multiple cell types to regulate cell growth, proliferation, and differentiation. The earliest evidence for a potential role of the JAK/STAT pathway in myogenic differentiation was provided by Guillet-Deniau et al. in 1997, where they showed serotonin-mediated activation of JAK-STAT was coupled to the upregulation of myogenin expression in myogenic progenitor cells [66,67]. The effects of JAK/STAT pathway are non-uniform, as different JAK/STAT isoforms can exert varied effects on muscle cell function. One investigation revealed the repressive effects of JAK1-STAT1-STAT3 on the expression of myogenic regulators, MyoD and MEF2C, and their target gene, *MHC* (myosin heavy chain). Additionally, JAK1-depleted myoblasts showed higher expression of CDK2-inhibitors (p21Cip1 & p27Kip1), thereby resulting in a reduced proliferation rate. This implies that the JAK/STAT pathway functions to promote the proliferation of myoblasts whilst preventing premature activation of the myogenic differentiation [68,69]. Interestingly, the other members of the JAK-STAT family, namely JAK2-STAT2-STAT3, operate in a pro-differentiation manner and stimulate myogenic differentiation. Knockdown and/or inhibition of JAK2, STAT2, and STAT3 in mice myoblasts led to downregulation of MyoD and MEF2 and their target genes *MHC* and *myogenin* [70]. Together, these data suggest the diverse role of the JAK/STAT pathway in myogenic differentiation. 

One of the key inducers of JAK/STAT pathway is IL-6, the surge of which activates STAT3 thereby promoting muscle growth. However, sustained release of IL-6 has been correlated with the expression of skeletal muscle ubiquitin E3 ligases in CIC. Additionally, STAT3 further potentiates muscle wasting by stimulating CCAAT/enhancer binding protein (C/EBPδ), leading to increased expression of myostatin, MuRF1, and atrogin1, thereby activating the ubiquitin proteosomal system in skeletal muscle cells [71]. STAT3-mediated muscle atrophy stems not just from IL-6, but is also induced by the FoxO transcription factor that specifically triggers and activates the muscle catabolic system [72]. Pharmacological inhibition of components of JAK/STAT has been shown to reduce muscle mass loss in cachectic mice, showing the JAK/STAT pathway to support modulation as a potential therapeutic target for cancer-induced cachexia [73].

### 2.6. Cachexia, Zinc Homeostasis, and Satellite Cell Differentiation

Recent studies point towards a connection between zinc ions and zinc transporters with cancer as well as with CIC. Increased expression of the zinc ion transporter ZRT- and IRT-like protein 4 (ZIP4) in pancreatic adenocarcinoma was associated with increased cell proliferation and tumor growth of pancreatic cancer cells. The elevated intracellular zinc levels induced by ZIP4 thus contributed to the progression and pathogenesis of pancreatic cancer [74]. Furthermore, pancreatic carcinoma-related ZIP4 induced the release of extracellular vesicles containing heat shock protein (HSP70 and HSP90) and thus activated p38MAPK, which in turn induced MAFbx and UBR2 expression in myotubes, leading to muscle atrophy [75]. Very interestingly, another metal-ion transporter ZIP14 was shown upregulated in cachectic skeletal muscles of mice and in human patients with metastatic cancer [76]. Higher levels of ZIP14 increased intracellular zinc levels and reduced the expression of myogenic transcription factors MyoD, Mef2c, and Myf5. Consequently, differentiation of muscle progenitor cells was blocked. Altered zinc homeostasis and increased ZIP14 expression were also observed in pancreatic and breast cancer related cachexia [77]. Besides ZIP proteins, the surge of pro-inflammatory cytokines, e.g., TNFα, IFNγ, and IL-1β, in cancer activates a network of transcription factors, including NF-κB and STAT1, that deregulate MyoD expression, thereby inhibiting muscle differentiation. Additionally, the cytokine-induced transcriptional machinery triggers inducible nitric oxide synthase (iNOS) expression, leading to accumulation of peroxynitrite, which is believed to cause destabilization and decay of *MyoD* transcript in the cytosol, consequently decreasing MyoD protein level. This mechanism of MyoD deregulation would not only affect functional skeletal myotubes, but also progenitor satellite cells, ultimately resulting in poor regenerative ability of skeletal muscle in cachectic patients [78,79,80]. Metabolic reorientation benefits the growth of cancer cells at the expense of muscle tissue. 

**Figure 1:**
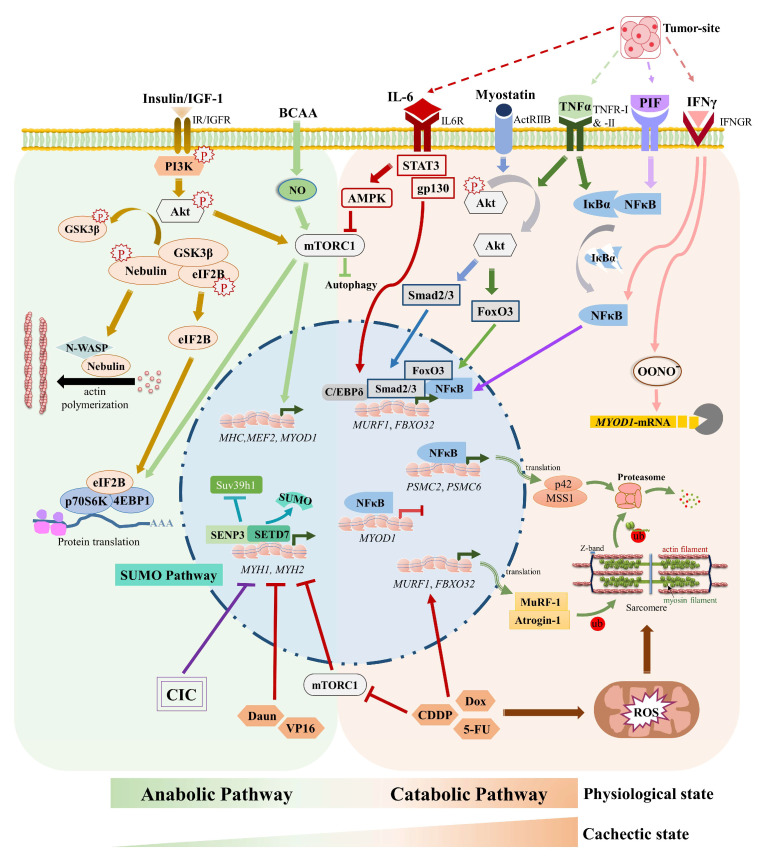
Pathways involved in anabolic and catabolic regulation of healthy and cachectic skeletal muscle. Anabolic regulation of skeletal muscle development triggered by Insulin, IGF-1 and BCAA leads to activation of Akt and mTOR signaling to promote expression of muscle proteins and polymerization of actin filaments. CIC is promoted by the activation of E3 ubiquitin ligase *MURF1* and *FBXO32* transcription in IL-6-, TNFα-, Myostatin-, IFN-γ, and PIF-mediated manner. Furthermore, transcriptional activation of proteasome ATPase subunits Mss1 and p42 is NF-κB-dependent. IFN-γ signaling leads to degradation of *MYOD1*-transript. IL-6/STAT3/AMPK signalling deactivates mTORC1 in CIC. Chemotherapy-triggered cachexia inhibits mTOR-mediated muscle protein synthesis, and activates MuRF1 & Atrogin1-mediated degradation of sarcomeric proteins. Furthermore, chemotherapeutics like Daun inhibit myosin heavy chain expression via SENP3 degradation. Pointed arrows indicate upregulation; T shaped arrows indicate downregulation. Note that not all the known signaling pathways and interconnections among them are represented in the above schematic. ActRIIB: Activin receptor type-2B; Akt: Protein kinase B; AMPK: AMP-activated protein kinase; BCAA: Branched amino acids; CDDP: Cisplatin; C/EBPδ: CCAAT/enhancer-binding protein delta; CIC: Cancer-induced cachexia; Daun: Daunorubicin; Dox: Doxorubicin; eIF2B: Eukaryotic translation initiation factor 2 subunit 2; FBXO32: F-box only protein 32/Atrogin-1; FoxO32: Forkhead box protein O3; gp130: skeletal muscle glycoprotein 130; GSK3β: Glycogen synthase kinase-3 beta; IκBα: NFκB inhibitor α; IFNγ: Interferon gamma; IFNGR: IFNγ receptor; IGF1: Insulin-like growth factor-1; IGFR: Insulin-like growth factor-1 receptor; IL-6: Interleukin-6; IL6R: IL-6 receptor; IR: Insulin receptor; MEF2: Myocyte enhancer factor 2; mTORC1: Mammalian target of rapamycin complex 1; MuRF-1: Muscle-specific RING finger protein 1; MYH1: Myosin heavy chain 1 (IIx/d); MYH2: Myosin heavy chain 2 (IIa); MyoD1: Myoblast determination protein 1; NFκB: Nuclear factor of kappa light polypeptide gene enhancer in B-cells; NO: Nitric oxide; N-WASP: Actin nucleation-promoting factor WASL; OONO^−^: Peroxynitrite anion; P: phosphate group; PIF: Proteolysis inducing factor; PI3K: Phosphoinositide 3-kinase; PSMC2/Mss1; 26S proteasome regulatory subunit 7; PSMC6/p42: 26S proteasome regulatory subunit 10B; p70S6K: Ribosomal protein S6 kinase beta-1; ROS: Reactive oxygen species; SENP3: Sentrin-specific protease 3; SETD7: Histone-lysine N-methyltransferase SETD7; Smad2/3: Mothers against decapentaplegic homolog 2/3; STAT3: Signal transducer and activator of transcription 3; SUMO: Small ubiquitin-related modifier; Suv39h1: Histone-lysine N-methyltransferase SUV39H1; TNFα: Tumor necrosis factor alpha; TNFR: TNF receptor; ub: Ubiquitin; VP16: Etoposide; 4EBP1: Eukaryotic translation initiation factor 4E-binding protein 1; 5-FU: Fluorouracil.

Although various signaling pathways that have been discussed above are known to be associated with CIC (Figure 1), the role of the SUMO pathway in the regulation of muscle function and CIC is far from understood. In the following section, we will mainly focus on the recent developments on this issue.

## 3. SUMO Pathway, Muscle Physiology, and Cachexia

### 3.1. The SUMO Pathway

Small ubiquitin-like modifiers or SUMOs (~12 kDa in size) are members of the ubiquitin-like protein family that can be covalently conjugated to a large number of proteins via an ATP-dependent SUMOylation process. This relatively new biological pathway was identified in the late 1990s, as the SUMO conjugation of RANGAP1 (a GTPase activator for the protein RAN) among the first SUMO-modified substrate proteins was reported [81,82] to enables nucleocytoplasmic transport. The SUMO pathway, similar to the ubiquitin pathway, comprises of the SUMO activating E1 enzyme, a conjugating E2 enzyme Ubc9, and a small group of E3 ligases. SUMO1 and highly similar SUMO2 and SUMO3 are the major functionally relevant isoforms expressed by mammalian cells. An elegant mass spectrometry-based study showed a higher abundance of SUMO2 proteins in mammalian cells (8.8 million copies/cell) and a relatively low level of SUMO1 (~410,000 copies) and SUMO3 (~64,000 copies) proteins [83]. With these numbers, SUMO has been designated as the 20th most abundant protein in mammalian cells. The SUMO-specific isopeptidases or SENPs can remove SUMO moieties from target proteins and render this process reversible [84]. By altering the molecular surface of substrate proteins, SUMO can alter higher-order protein assemblies, their subcellular distribution, and the fate of target protein function. Current estimates from quantitative proteomic studies suggested that more than 20% of human proteome undergoes SUMO modification [22], underscoring the fundamental impact of SUMOylation in cell function. Genetic ablation studies showed that SUMO modification is indispensable during the embryonic as well as postnatal development of mammals [85,86]. SUMO-regulated molecular events occur predominantly in the nucleus, where SUMOylation controls processes of transcription, RNA processing, the DNA damage response, cell cycle progression, and dynamics of specialized nuclear sub-domains such as PML-Nbs [87,88]. These modes of regulation have diverse functions in various cellular physiological processes, such as cell fate determination, nuclear organization, and the maintenance of pluripotency, immune responses, cancer cell dynamics, cardiovascular functions, mitochondrial metabolism, and proteostasis. The SUMO pathway acts as a molecular stress sensor and plays a key role in a process known as liquid–liquid phase separation (LLPS), which generates bio-molecular condensates or membrane-less organelles [89,90]. One characteristic feature of the SUMO-mediated regulation is that the SUMO-modified species represents an extremely low fraction of its total substrate protein. Despite the low SUMOylated protein fraction levels, the SUMO pathway exerts significant changes in cell function. This paradox was explained by the SUMO pathway’s “group modification” strategy, i.e., the co-modification of large sets of functionally and topologically connected substrate proteins mediated by the SUMO pathway [91]. Further details on SUMOylation and cell physiology are reviewed elsewhere [92,93,94,95]. 

### 3.2. SUMOs in Muscle Physiology

The SUMO pathway is essential in both striated and smooth muscles. Besides, the SUMO pathway operates at various levels of muscle physiology, such as myogenic differentiation, sarcomere assembly, muscle contraction, and muscle carbohydrate metabolism, etc. One of the first seminal observations of SUMOs in muscle physiology elucidated the role of the SUMO E2 conjugating enzyme mUbc9 in regulating glucose transport in skeletal muscle cells [96]. By using a yeast two-hybrid screening system, mUbc9 (or Ubc9) was found to be interacting directly with GLUT4 (Glucose transporter type 4) and GLUT1. In insulin-sensitive muscle tissues, GLUT1 is largely responsible for basal glucose transport and GLUT4 is accountable for rapid glucose uptake in response to insulin. mUbc9 was shown to regulate GLUT1 and GLUT4 protein levels as overexpression of mUbc9 in L6 skeletal muscle cells lowered GLUT1 levels, resulting in decreased basal glucose transport. Contrary to GLUT1 levels, mUbc9 overexpression increased GLUT4 protein abundance and upregulated glucose uptake upon insulin stimulation. GLUT4 was reported as SUMOylated [97] and a dominant-negative mUbc9 catalytically inactive variant displayed opposite effects on GLUT1/4 levels and glucose uptake compared to the wild-type mUbc9. In line with this report, another study observed a correlation between increased SUMOylation and GLUT4 stability upon the action of sortilin, a protein implicated in the formation of insulin-responsive GLUT4 storage vesicles [98]. The involvement of the SUMO pathway was also described in fatty acid metabolism in skeletal muscle. Treatment of skeletal myotubes with saturated fatty acids, like palmitate, led to NF-κB-mediated upregulation of transcription and protein level of the SUMO-specific isopeptidase 2 (SENP2) [99]. This increased SENP2 level promoted deSUMOylation and the recruitment of peroxisome proliferator-activated receptor delta (PPARδ) to the promoters of the genes regulating fatty acid oxidation (FAO), including carnitine-palmitoyl transferase-1 (*CPT1b*) and long-chain acyl-CoA synthetase 1 (*ACSL1*). Later, the SENP2-dependent pathway was shown to be responsible for a slow, prolonged increase in FAO [100]. Through this regulation, SENP2 ultimately alleviated high-fat diet-induced obesity and insulin resistance. 

Another pioneering study showed SUMO modification of the MEF2 family of myogenic transcription factors Mef2A, Mef2C, and Mef2D [101,102]. The class IIa deacetylases such as HDAC4, 5, 7, and HDAC 9 isoforms, enhanced the SUMOylation of MEF2, and the same was reduced by the SUMO isopeptidase SENP3. Enhanced SUMOylation established a repressive effect on MEF2 transcriptional activity. The differentiation signal relieved MEF2 SUMOylation through activation of the MEK5 and ERK5 pathways, which stimulated MEF2 transcriptional activity. The phosphorylation-dependent SUMOylation motif (PDSM) composed of a SUMO conjugation consensus site and an adjacent phosphorylation site proline (ψKxExxSP) is observed in various substrates including heat-shock factors that undergo phosphorylation-dependent SUMOylation through this conserved motif [103]. Interestingly, phosphorylation of MEF2C at Serine 396 stimulates its SUMOylation at lysine residue 391. This was proposed to recruit co-repressors and explains the mechanism of how SUMOylation inhibits the transcriptional potential of MEF2. In addition, interfering with SUMO pathways by knocking down Ubc9 severely compromised myogenic differentiation. However, the localization or the activation of key myogenic regulators, such as MyoD and myogenin, remained unchanged after Ubc9 knockdown [104]. Similarly, a relatively recent study also reported inhibition of myogenic differentiation when myoblasts were treated with 2-D08, an inhibitor of SUMOylation [105]. Typically, 2-D08 prevents the transfer of activated SUMO from the E2 to the substrates without affecting the SUMO E1 enzyme. In contrast to Ubc9 knockdown-mediated inhibition of myogenic differentiation, 2-D08 perturbed myogenesis by activating Erk1/2 and decreasing myosin heavy chain, MyoD, and myogenin expression. Erk1/2 kinase belongs to the mitogen-activated protein kinase (MAPK) pathway that promotes skeletal muscle cell proliferation but negatively regulates myogenic differentiation [106]. Despite the different approaches in these studies, a common observation was that the inhibition of the SUMO pathway intrinsically affects myogenesis, consequently affecting muscle cell function. 

**Figure 2:**
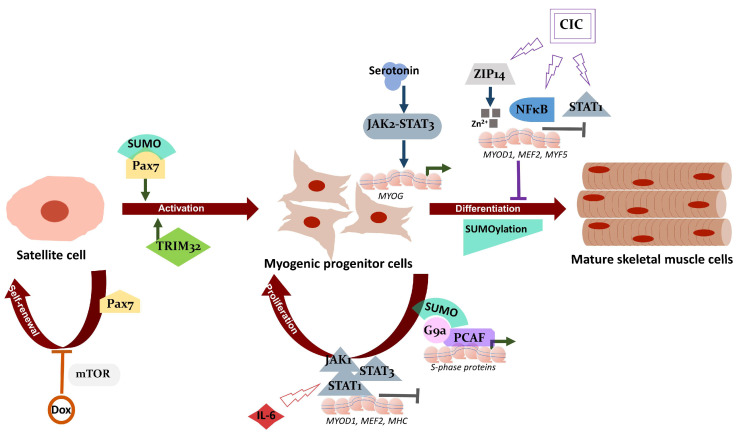
Influence of cachexia on muscle regeneration and myogenesis. Pax7 maintains self-renewal ability of satellite cells, while TRIM32 and SUMOylation of Pax7 regulate the activation of satellite cells to form progenitor myoblast cells during muscle regeneration. Chemotherapeutics like Dox inhibit satellite cell proliferation via the mTOR pathway, thereby reducing the stem cell pool for muscle regeneration. Upon stimulation by serotonin, the JAK2-STAT3 network is activated, leading to expression of myogenin, a key determinant of myogenesis. On the other hand, IL6-led JAK1-STAT3-STAT1 activation allows downregulation of MyoD1, MEF2 and MHC, which inhibits premature myogenic differentiation and maintains sufficient number of myoblasts. The myogenic progenitor pool is additionally maintained by the expression of S-phase proteins that are regulated by SUMO-modified-G9a and PCAF complex. The surge of pro-inflammatory cytokines in cancer-induced cachexia activates NF-κB and STAT1 signaling that deregulates muscle differentiation through MyoD suppression. Additionally, CIC upregulates ZIP14 thereby leading to accumulation of zinc ion and reduced expression of key myogenic factors. Global SUMOylation is reduced during myogenesis, suggesting a strict regulation and interconnection of the various signaling networks in the process of myogenic differentiation and muscle regeneration. Pointed arrows indicate positive regulation; T shaped arrows indicate negative regulation. CIC: Cancer-induced cachexia; Dox: Doxorubicin; Erk1/2: Extracellular signal-regulated kinase 1/2; G9a: Histone-lysine N-methyltransferase EHMT2; IL-6: Interleukin 6; JAK1: Janus kinase 1; JAK2: Janus kinase 2; MHC: Myosin heavy chain; MEF2: Myocyte-specific enhancer factor 2; mTOR: Mammalian target of rapamycin; MYOD1: Myoblast determination protein 1; MYOG: Myogenin; MYF5: Myogenic factor 5; NFκB: Nuclear factor of kappa light polypeptide gene enhancer in B-cells; Pax7: Paired box protein Pax-7; PCAF: Histone acetyltransferase PCAF; STAT1: Signal transducer and activator of transcription 1; STAT3: Signal transducer and activator of transcription 3; SUMO: Small ubiquitin-related modifier; TRIM32: E3 ubiquitin-protein ligase TRIM32; ZIP14: zinc ion transporter ZRT- and IRT-like protein 14; Zn: Zinc.

Another prominent example in this context is the SUMO modification of the paired box protein (Pax7). Pax7 is expressed in muscle stem cell progenitors and is necessary for the self-renewal and activation of adult satellite cells. Pax7 regulates various genes, including inhibitors of DNA binding 3 (*ID3*) [107]. SUMO conjugation on lysine 85 (K85) of Pax7, but not on highly conserved K85 of Pax3, is necessary for the differentiation potential of the myoblasts [108]. Similarly, SUMO1 modification of the co-repressor G9a histone methyltransferase was essential in promoting the proliferation of the myoblasts [109]. SUMOylation of G9a favored its interaction with the transcriptional activator PCAF (p300/CBP-associated factor) and increased the association of PCAF on E2F1 target genes as required for S-phase progression. This mechanism ensures proliferation and preserves the undifferentiated state of progenitor myoblast cells (Figure 2).

In addition to regulating muscle progenitor cell proliferation and differentiation process, SUMO’s role in fully differentiated mature muscle is interesting yet complex. Skeletal muscle fibers in mammals are broadly grouped as type 1 or slow-twitch and type 2 or fast-twitch fibers [110]. Depending on the presence of myosin heavy chain protein isoforms, the muscle fibers are further categorized as types 2A, 2X/D, and 2B. Furthermore, the fiber types display discrete metabolic signatures. While muscle fiber types 2X and 2B fibers depend on glycolytic metabolisms as the primary ATP source, oxidative metabolism (i.e., oxidation of glucose) is predominantly used in types 1 and 2A fibers to produce ATP. Skeletal muscle can dynamically change the fiber type compositions depending on a specific environmental challenge. This can be in response to a type of physical training or under pathological conditions as in neuromuscular disorder. The fast or rapid movements are mainly driven by the glycolytic type 2 fast-twitch fibers containing fast myosin isoform. The slow-twitch type 1 oxidative fiber, which expresses slow myosin isoforms, supports load-bearing capacity or maintains the body posture. The mechanism of fiber-type switches in response to muscle activity has been under intense investigation. Only very recently, it was shown that muscle tissue responds to mechanical loading and unloading by not only changing myosin isoform, but also by altering SUMOylation levels of target proteins in the muscle tissue [111]. A comprehensive study with nine different mammalian muscle tissues exhibited a distinct group of SUMO enzymes and SUMOylated proteins present in distinct muscle fiber types with different modes of metabolism [112]. *Musculus soleus* (slow-oxidative type I muscle) displayed a greater number of SUMO1-conjugated proteins than SUMO2 conjugates. Compared to the slow fibers, the overall SUMO1 conjugates were significantly low in fast-glycolytic type II muscle fibers. The amount of SUMO1 conjugates varied among the type II fiber types. The diverse activity of muscles can be attributed to varied expression of SUMO machinery, as distinct muscle types showed a strong correlation between transcript and protein abundance of distinct components of the SUMO pathway. Although it was speculated that the muscle-type-specific diverse SUMO signature could have arisen due to their distinct embryological origin as well as different metabolic properties, the reason underlying SUMO isoform specification in distinct muscle types is still not clear. One intriguing aspect of this study was a rapid alteration of global SUMOylation observed in response to muscle activity, particularly under mechanical unloading of muscle. In the tail-suspended ambulatory rat, soleus muscle underwent a phenotype shift to glycolytic fibers with a concomitant increase in total SUMO1 and SUMO2-modified proteins, decreases in SUMO isopeptidases, SENP2, SENP5, and SENP6 levels, without changes in the total protein poly-ubiquitination. Again, the consequence of this event is not yet clear. One possible explanation for this is that by responding to changed muscle activity, the SUMO pathway helps muscle adaption and protects the muscle’s contractile apparatus from immediate degradation. Taken together, the SUMO system exhibits distinct footprints with implications in the regulation of muscle physiology (Figure 1 and Figure 2).

### 3.3. SUMOs in Muscle Atrophy including Cachexia

In response to physiological and pathological signals, skeletal muscles adapt and undergo various degrees of remodeling. The adaptive mechanisms ensure muscle homeostasis by regulating muscle mass and performance through a fine balance between muscle protein synthesis and degradation. Muscle regeneration is another critical factor in maintaining muscle mass. Cachexia signaling (in both CIC as well as chemotherapy-induced cachexia) causes muscle atrophy by shifting this delicate balance toward increased protein degradation, particularly of myofibrillar proteins (Figure 1). The key muscle-specific E3 ubiquitin ligases, MuRF, atrogin1 and UBR2, have been classified as major contributors regulating skeletal muscle mass through the ubiquitin proteasome pathway (UPS). Degradation of myofibrillar proteins including myosin heavy chain (MHC) is reported in cachexia [29]. Despite the central role of MuRF family protein in cachexia-induced muscle loss, *MuRF1* knockout only partly protected mice from denervation-induced muscle loss. *MuRF1* knockout mice were not spared from either microgravity- or fasting-induced muscle atrophy [28,113]. Moreover, mice with genetic ablation of MAFbx exhibited neither higher muscle myofiber area nor higher muscle hypertrophy than WT mice in response to functional overload [114]. These seminal studies imply the possible involvement of novel mechanisms and mediators beyond MuRFs/MAFbx in regulating muscle mass that would be crucial for understanding the cachectic condition. 

The SUMO pathway is emerging as one of the previously unknown mediators of muscle atrophy including CIC. The connection of the tripartite motif-containing protein 32 (TRIM32) family with SUMO E3 ligase PIAS4 was among the pioneering studies linking SUMOs with muscle atrophy. Unlike MuRF1/TRIM63, TRIM32 is another tripartite motif family E3 ubiquitin ligase expressed ubiquitously in the skeletal muscle [115]. Mutations in *Trim32* are associated with limb-girdle muscular dystrophy type 2H (LGMD2H). Interestingly, a detailed investigation by Kudryashova et al. [115] revealed that TRIM32 is dispensable in muscle atrophy. However, TRIM32 plays an important role in muscle growth after disuse atrophy. By generating TRIM32-deficient primary myoblasts, they found that TRIM32 regulates satellite cell proliferation and myogenic differentiation and is necessary to prevent premature senescence of myogenic cells. TRIM32 deficient myoblasts exhibited an increased level of PIAS4 together with global SUMOylation and other replicative senescence mediators, such as heterochromatin protein 1 (HP1γ) and p53, which are all typical features found in sarcopenia and type II fiber atrophy associated with myopathy and LGMD2H. Thus, compared to wild-type muscles, TRIM32 deficient muscles had increased PIAS4 levels and substantially fewer activated satellite cells. Moreover, *Trim32^–/–^* muscles exhibited features of early sarcopenia signs, such as selective type II fast fiber atrophy. These results imply that premature senescence of muscle satellite cells is perhaps among other pathogenic routes of muscular dystrophy associated with SUMO systems. Whether the same mechanism is also true in CIC and other related muscle atrophies remains undetermined. 

An intricate connection between SUMO and cachexia emanated from the findings of the SUMO isopeptidase SENP3’s association with muscle cells organization and function [116]. Under normal conditions, temporal epigenetic regulation of *myosin heavy chain* genes, namely *MyHC-IId* and *MyHC-IIa*, by SENP3 was found important for proper sarcomere organization and muscle cell contraction. SENP3 expression itself was temporally upregulated during myogenic differentiation and the association of SENP3 with the histone methyltransferase SETD7 ensured proper expression of *MyHC-IId/a.* Mechanistically, SENP3 interacted with SETD7. An SENP3 mediated SETD7 deSUMOylation event was necessary for proper chromatin targeting of SETD7 and prevented the association of repressive histone methyltransferase Suv39h1 on *myosin heavy chain* gene (*MyHC-IIa/d*). This: (1) promoted a transcriptionally competent epigenetic milieu with improved mono-methylation on lysine 4 of histone 3 (H3K4me1) and reduced transcriptional suppressive epigenetic H3K9me3 marks, and (2) stimulated the association of transcriptionally active RNA Polymerase II (RNA Pol II) on *MyHC-IId* promoter. This SUMO-mediated regulation was found to be crucial for the expression of *myosin heavy chain*, correct sarcomere assembly, and proper contractile ability of muscle cells. In cachexia, particularly in denervation-induced cachexia in amyotrophic lateral sclerosis (ALS), the SENP3-dependent transcriptional pathway was targeted. Cachexia signaling destabilized the SENP3 protein level. As a result, SETD7 chromatin residency was reduced. Subsequently, *MyHC-IId/a* gene acquired more H3K9me3 and less H3K4me1 marks and impeded the loading of RNA Pol II on the *myosin heavy chain.* Consequently, a severe downregulation of *MyHC-IId/a* was observed as a primary underlying cause of sarcomere disarray, another characteristic feature of cachectic muscle. One interesting aspect of this finding was that SENP3-goverened regulatory processes were specific towards genes coding major *myosin heavy chain* isoforms *MyHC-IId* and *MyHC-IIa*. Other sarcomeric genes encoding proteins of contractile apparatus, such as *actinin, titin, troponin* etc., remained unaffected by SENP3´s regulation. 

Another recent finding from our group unraveled a link of SUMO pathway with chemotherapy-induced cachexia [19]. Since CIC leads to loss of muscle mass and the doses of chemotherapeutic drugs are based on body surface area, cancer patients with altered muscle surface area are prone to receive improper doses of treatments, thus becoming more susceptible to chemotherapy-associated side effects, and grow too weak to tolerate further therapies [117]. Reports, including our own studies, showed that apart from targeting cancers, chemotherapeutic drugs induce cachexia. Thus, in addition to cancer-induced cachexia, chemotherapy-triggered cachexia ultimately leads to a profound loss of muscle mass and function. Various chemotherapeutic drugs, including doxorubicin (DOX), cisplatin (CDDP), 5-fluorouracil (5FU), etoposide, etc., are known to induce cachexia through various modes [118]. These drugs can promote systemic inflammation via the central nervous system to promote an adaptive illness response and induces the release of glucocorticoids and pro-inflammatory cytokines such as TNFα, IL6, etc. [119]. This in turn can directly induce skeletal muscle atrophy via the activation of a pro-catabolic transcription program as we have described before. Another observed effect of the drugs included altered dynamics of mitochondrial metabolism following the drug treatment, whereby it can directly stimulate reactive oxygen species (ROS) production via affecting NADH dehydrogenase/complex I of the mitochondrial electron transport chain. Elevated ROS levels- particularly hydrogen peroxide (H_2_O_2_) can promote oxidative damage of contractile proteins, especially actin and myosin [120] (Figure 1). Chemotherapeutic agents such as DOX were shown to interfere with the mTOR pathway and inhibit satellite stem cell proliferation and thus impairing the regenerative capacity of muscle as the satellite stem cell pool decreases [121]. DOX also activated the p53-p21-REDD1 signaling axis toward establishing a chemotherapy-induced cachexia [122]. REDD1 (Regulated in Development and DNA Damage Response 1), a stress-response protein, represses mTORC1 towards causing muscle wasting, and loss of REDD1 prevents chemotherapy (carboplatin)-induced cachexia in mice [123]. We showed that specific chemotherapeutic agents, through alteration of the SUMO pathway, can cause chemotherapy-induced cachexia [19]. A comparative analysis of various classes of chemotherapeutic drugs frequently used in clinics showed their distinct modes of action on muscle cells. For example, daunorubicin (Daun) and etoposide (VP16) treatment led to severe sarcomere disarray in C2C12 muscle cells as well as satellite cell-derived primary muscle cells. In-depth analysis provided further information on the molecular mechanisms of Daun and VP16 modulated epigenetics processes in *myosin heavy chain* genes by targeting SUMO isopeptidase SENP3-regulated transcriptional processes. Daun and VP16 destabilized SENP3 levels in myotubes and impaired chromatin targeting of SETD7 and its association of SETD7 with histone acetyltransferase p300. Under physiological conditions, SENP3 promotes the interaction of these two epigenetic regulators. Drugs affected the interaction between SETD7 and p300, leading to a changed epigenetic signature on *myosin heavy chain* genes, characterized by a diminished level of pan histone 3, histone 4 acetylation, and H3K4me1.

In summary, both CIC and chemotherapy-induced cachexia require further investigation for the presence of other possible pathways leading to cachexia. SUMO-dependent pathways appear to be among the ones with important implications to understand the development of cachexia.

## 4. Current Treatment Strategies

In the past, several treatment strategies were examined to revert CIC, ranging from nutritional support to exercise and pharmacological intervention. A complete reversal of cachexia, however, has not been achieved with any single or combined treatments. Current suggested treatment guidelines mainly focus on multimodal, combinatorial strategies. In this section, we discuss these strategies for mice CIC models as well as human CIC patients. 

The nutritional supplement is one line of strategy. The application of omega-3 poly-unsaturated fatty acid eicosapentaenoic acid (EPA) in a clinical trial for cachectic patients suffering from gastrointestinal and lung cancer has shown a trend toward improved lean body mass (LBM) [124]. In a different study, EPA in combination with another omega-3 poly-unsaturated fatty acid, docosahexaenoic acid (DHA), and Vitamin D3 resulted in fewer adverse events compared to the control group, although without changes in body weight, LBM, or hand grip strength [125]. Beta-hydroxy-betamethylbutyrate (HMB), a metabolite of the essential amino acid leucine, is another dietary supplement that alleviated the protein imbalance by boosting protein synthesis and reducing protein catabolism. It was even more potent when applied in combination with EPA in a murine adenocarcinoma 16 (MAC16) tumor mouse model of CIC [126]. HMB in combination with arginine and glutamine increased the body weight of solid tumor patients in a double-blind, placebo-controlled study [127]. Recently, Prado and colleagues put together a systematic review on HMB supplementation in cancer patients, suggesting a positive effect on muscle mass and function, reduction in hospitalization, and increased survival [128]. Moreover, oral supplementation of 4000 mg of curcumin per day in cachectic patients with head and neck cancers resulted in an increased muscle mass without any detectible changes in basal metabolic rate or handgrip strength [129]. Nutritional supplementation in the form of energy, namely fat and protein-rich flat bread (‘Improved Atta’), over a six-month period in cachectic women in palliative care increased both body weight and fat mass, without any determined report on the change of muscle mass [130]. Besides, several orexigenic drugs were also tested to increase the food intake of cachectic patients and improve the loss of LBM. One such instance is the ghrelin receptor agonist anamorelin, which increased body mass, body weight, and appetite in multiple 12-week trials in cachectic patients with non-small cell lung cancer (NSCLC) without changes in any fitness test [131,132], which was maintained for a further 12 weeks [133]. In a cachectic mouse lung cancer model, anamorelin in combination with the activin A ligand trap (ActRIIB) not only increased appetite and LBM, but also led to an increased survival rate. However, the same results could not be achieved in the human clinical trials [134].

Apart from nutritional supplements, exercise intervention regimes such as endurance, resistance, or combined exercises were studied both in tumor-bearing animal models as well as in cachectic cancer patients. The idea of this intervention was to investigate whether various exercise regimens can prevent or delay the onset of cachectic muscle wasting by enhancing protein translation and mitochondrial metabolism and reducing the increased protein catabolism. A combined exercise protocol in C26-bearing mice prior to and after tumor implantation resulted in increased muscle weight and strength by reducing levels of the autophagy marker LC3B-I/II ratio [135], while improving redox homeostasis and increasing mitochondrial mass [136]. In rats bearing Walker-256 tumors, resistance training attenuated tumor-induced muscle oxidative stress and muscle damage [137]. Even a low-intensity endurance exercise prevented loss of muscle mass and strength in cachectic rats bearing AH130 Yoshida ascites hepatoma cells [138]. Among the possible mechanisms, it was shown that the exercise regimens could suppress muscle degradation pathways, e.g., the ubiquitin-proteasome function, and suppress AMPK by increasing AMPK phosphorylation.

One major problem in exercise therapy is the lack of motivation for exercise among CIC patients. To circumvent the problem, an interesting idea of ‘exercise-mimetic’ agents has been under investigation. Trimetazidine and 5-aminoimidazole-4-carboxamide ribonucleoside (AICAR), used as an ‘exercise-mimetic’ in CIC induced myogenesis, increased grip strength and partially restored muscle cross-sectional area in C26-bearing mice [139,140]. In response to exercise or exercise mimetics, AMPK inhibited the mammalian target of rapamycin (mTOR) signaling and activated FoxO3a towards promoting skeletal muscle lysosomal autophagy flux to restore the muscle homeostasis [141,142]. Both excessive activation of autophagy and inhibition of lysosome-dependent degradation aggravate muscle wasting and are linked to myopathies [46]. Despite all these encouraging studies, the effectiveness of exercise on LBM in cachectic cancer patients in clinical trials remains rather limited [143]. Moreover, increasing protein intake and nutritional supplement did not affect either the survival rate or the body weight in chemotherapy-treated cancer patients [144]. Currently, there is no consensus on the type of exercises with the optimal effect on muscle mass and function in patients. A combination of both resistance and endurance exercise was suggested [145]. However, this should always be personalized to the needs and physical capacities of the patient. Along the lines of personalized treatments, nutritional support shows a higher potential towards improved cachexia management.

Altogether, a combination of exercise and dietary supplements with high-energy formulas and protein anabolism-promoting agents, e.g., omega-3-fatty acids, can aid in maintaining or building muscles.

## 5. Conclusions

Cancer-induced cachexia is a result of complex metabolic alterations, sarcomeric protein degradation, depletion of the satellite cell population, and reduced myogenic differentiation. Muscle wasting and consequent dysfunction affect the regular activity of patients suffering from cancer and are even responsible for increased mortalities. Limited success using the above-mentioned treatment strategies means that there is an unmet need for new drugs and potential targets to effectively improve this debilitating condition. Cachexia affects multiple organ functions and thus it demands further in-depth studies to classify its various stages of development. In this context, CIC models of mice, which can be monitored at different stages of disease progression [76], might be instrumental to investigate the disease-specific molecular markers and their interplay with other established signaling pathways in muscle. The development of engineered human skeletal muscle cells derived from human pluripotent stem cells could further enhance our understanding of muscle development and diseases including cachexia [146]. Recently, another important pathway, the SUMO signaling pathway, has emerged as a key regulator in muscle cell organization and cachexia and is perceived as a potential target for therapeutic interventions [147]. The involvement of chemotherapeutic drugs in cachexia through functional alteration of components of the SUMO pathway provides more compelling evidence of an unconventional mechanism regulating cachexia signaling. Future studies aimed at developing and testing new small molecule inhibitors/activators regulating various components of the SUMO machinery may open new avenues for cachexia treatment strategies. Combined efforts involving knowledge of the underlying molecular mechanisms and novel treatment approaches will be key in the battle against CIC. Importantly, this knowledge may be useful to treat cachexia linked to other end-stage muscle-related illnesses.

## Data Availability

Not applicable.
